# The future of bioenergy

**DOI:** 10.1111/gcb.14883

**Published:** 2019-12-05

**Authors:** Walter V. Reid, Mariam K. Ali, Christopher B. Field

**Affiliations:** ^1^ David and Lucile Packard Foundation Los Altos CA USA; ^2^ Stanford Woods Institute for the Environment Stanford University Stanford CA USA

**Keywords:** bioenergy, bioenergy with CCS, biofuels, biomass, climate change, land scarcity, lock‐in, path dependency

## Abstract

Energy from biomass plays a large and growing role in the global energy system. Energy from biomass can make significant contributions to reducing carbon emissions, especially from difficult‐to‐decarbonize sectors like aviation, heavy transport, and manufacturing. But land‐intensive bioenergy often entails substantial carbon emissions from land‐use change as well as production, harvesting, and transportation. In addition, land‐intensive bioenergy scales only with the utilization of vast amounts of land, a resource that is fundamentally limited in supply. Because of the land constraint, the intrinsically low yields of energy per unit of land area, and rapid technological progress in competing technologies, land intensive bioenergy makes the most sense as a transitional element of the global energy mix, playing an important role over the next few decades and then fading, probably after mid‐century. Managing an effective trajectory for land‐intensive bioenergy will require an unusual mix of policies and incentives that encourage appropriate utilization in the short term but minimize lock‐in in the longer term.

## Graphical abstract

Energy from biomass plays a large and growing role in the global energy system. But because of the land constraint, the intrinsically low yields of energy per unit of land area, and rapid technological progress in competing technologies, land intensive bioenergy makes the most sense as a transitional element of the global energy mix, playing an important role over the next few decades and then fading, probably after mid‐century.

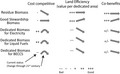

## 1 INTRODUCTION

Bioenergy plays a significant role in many scenarios for achieving the Paris goals of limiting climate change to well under 2°C (Rogelj et al., 2018). But a number of studies have challenged the greenhouse gas accounting, raising the concern that lifecycle emissions have been underestimated and that the ‘carbon debt’ associated with bioenergy often results in greater near‐term emissions than the fossil fuels being replaced. Other work emphasizes the prospect that growth in bioenergy could reduce food production and accelerate biodiversity loss (DeCicco & Schlesinger, 2018; European Academies Science Advisory Council, 2019; Searchinger, Wirsenius, Beringer, & Dumas, 2018).

In this article, we provide a framework for evaluating the role of bioenergy in climate mitigation over the next century. We focus this paper on ‘land‐intensive bioenergy’ by which we mean bioenergy from terrestrial plants (e.g., crops, trees, grasses) cultivated or harvested primarily for energy. We also discuss energy from plant residues or from material harvested as part of effective ecosystem management, which faces different constraints and opportunities. Land‐intensive bioenergy makes a meaningful contribution to the global energy system only at a spatial scale of hundreds of millions of hectares or larger, large enough to have significant trade‐offs with food production and biodiversity conservation. We argue that land‐intensive bioenergy is unlikely to be a major part of the energy mix by the end of the century. And, while bioenergy already plays a significant role in the current energy mix and will probably grow in coming decades, the costs associated with land‐intensive bioenergy are great enough and the technologies that can replace bioenergy are sufficiently promising, that land‐intensive bioenergy is not likely to be a significant part of the energy mix in the latter decades of the 21st century. For that reason, by mid‐century it is likely to be seen as a legacy fuel. Policies related to biomass energy development and deployment should aim to avoid lock‐in and open doors for the technologies that can replace bioenergy.

## 2 LONG‐TERM PROSPECTS FOR LAND‐INTENSIVE BIOENERGY

Bioenergy is a significant part of the energy economy, accounting for 9.5% of total primary energy supply and some 70% of renewable energy in use today (International Energy Agency, 2017b, 2019). More than half of this bioenergy involves the traditional use of biomass, mostly in households for cooking and heating but also within small industries (such as charcoal kilns and brick kilns). While there is considerable scope for improving the sustainability, efficiency, and health safety associated with the use of traditional biomass (Creutzig et al., 2015), this paper limits its focus to modern bioenergy because of its potential for significant growth in the coming decades.

Modern bioenergy (hereafter ‘bioenergy’) was responsible for half of all renewable energy consumed in 2017, providing four times the contribution of solar photovoltaic (PV) and wind combined (International Energy Agency, 2018). Most bioenergy delivers heat in buildings and industry, but bioenergy is also expected to account for 3% of electricity production and around 4% of transport energy demand in 2023 (International Energy Agency, 2018). Production of liquid biofuels for transportation grew at annual rates greater than 10% prior to 2010 but then slowed to 4% annual growth from 2010 to 2016. The annual average growth rate of bioenergy electricity capacity was 6.5% from 2010 to 2016 (International Energy Agency, 2017b). Over the period of 2018–2023, bioenergy (including liquid biofuels) is projected to account for 30% of the growth in renewable energy production (International Energy Agency, 2018).

**Figure 1:**
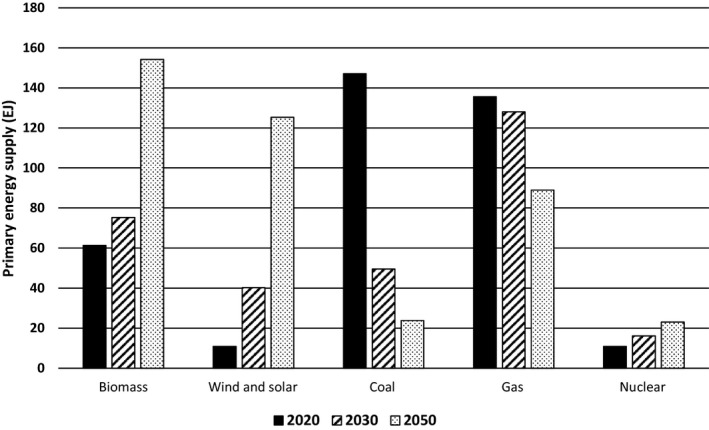
Median global primary energy supply based on eighty‐five 1.5°C pathways (combining low and high overshoot pathways). Under these pathways, net CO_2_ emissions decline from 38.5 Gt CO_2_/year in 2010 to a median of 29.1 Gt CO_2_/year in 2030 and 1.0 Gt CO_2_/year in 2050 (Rogelj et al., 2018)

Bioenergy features prominently in most recent scenarios for addressing climate change. The recent Intergovernmental Panel on Climate Change (IPCC) Special Report on *Global Warming of 1.5°C* reviewed eighty‐five 1.5*°*C pathways and found that biomass made up a median of 26% of primary energy (154 EJ/year) in 2050 (range from 10% to 54%), up from 10% in 2020 (Rogelj et al., 2018). Solar and wind, by comparison, account for a median of 22% of primary energy in 2050 (Figure 1). Many of the modeled pathways reviewed in that assessment project a continuing use of high levels of bioenergy without carbon capture and storage (CCS) through the end of the century, and a significant growth in bioenergy with CCS (BECCS; Rogelj et al., 2018). Some of the modeled pathways achieve a 1.5°C future without BECCS and with a reduced amount of bioenergy, but these are based on a substantial decline in overall energy demand (a reduction of 32% by 2050 compared to 2010 levels), significant changes in behavior (such as changes in diet), rapid technological progress, and a low global population.

The significant role for bioenergy in these scenarios, and particularly the significant role for BECCS, results from an optimistic framing that assumes large areas of land could be available for bioenergy production and that characterizes bioenergy as a relatively low‐cost and low‐emission source of energy that does not face the challenge of intermittency associated with renewables like solar and wind energy. As we discuss later in this paper, actual deployments of land‐intensive bioenergy are often not low‐emission energy sources. Furthermore, even if all bioenergy were carbon neutral, land‐intensive bioenergy is unlikely to be an economically attractive energy source post‐2050 because of technology innovation and market competition.

But, in our view, one of the most important limits to land‐intensive bioenergy is the availability of land. We do not expect land‐intensive bioenergy to be a competitive energy source over the long term precisely because it requires so much land, land is fundamentally fixed in quantity, and land is already a scarce resource. The combination of an increasing human population and an increasing appreciation of the conservation value of natural and mostly natural ecosystems points to growing scarcity with time, even if agricultural yields continue to increase. Studies that have attempted to estimate the technical potential for bioenergy production span a range of three orders of magnitude, from <50 to >1,000 EJ/year (Creutzig et al., 2015). Different views and assumptions about land availability, sustainability, and socioeconomic constraints underlie much of the variation in these estimates. For example, some studies identify a potential of up to 100 EJ/year for bioenergy from dedicated biomass plantations on marginal and degraded lands, but the question of just how much land is really unused and available is contested (Creutzig et al., 2015; Field, Campbell, & Lobell, 2008). And, demand for land will grow even without bioenergy. If agricultural productivity continues to increase at the pace that was achieved over the past 50 years and no land is allocated to bioenergy production, crop and pastureland will still need to expand 10% by 2050 (Searchinger, Waite, Hanson, & Ranganathan, 2018).

The conversion of natural habitats and ecosystems to managed landscapes, agriculture, and urban areas has already undermined ecosystem services that humanity depends upon and has placed one million species at the risk of extinction (Díaz et al., 2019; Reid et al., 2005). There is thus a need to protect remaining natural ecosystems, and, wherever possible, to restore lost or degraded ecosystem services from degraded lands or lands retired from food or fiber production. The need for conservation and restoration is largely incompatible with the large‐scale expansion of land‐intensive bioenergy. The IPCC concluded, for example, that at the scale needed for bioenergy, reforestation, and afforestation to make a meaningful contribution to emissions reductions and CO_2_ removal, the increased demand for land conversation would have adverse effects on desertification, land degradation, and food security (Arneth et al., 2019).

Because land is scarce, if we do need to use land to produce energy, we should use it as efficiently as possible. PVs, for example, can provide dramatically more efficient use of land for energy production. The amount of electricity that can be produced from a hectare of land using PVs is at least 50–100 times that from biomass (European Academies Science Advisory Council, 2019).

Despite its relatively inefficient use of land and the potential competition with other land uses, bioenergy plays a significant role in most energy scenarios past mid‐century for three reasons. First, unlike intermittent sources of energy, bioenergy can meet needs for baseload electrical power, a characteristic thought to be increasingly important as the existing fossil fuel‐based thermal capacity is retired. Second, applications in shipping and aviation require fuels with a high energy density, and biofuels can meet this criterion at relatively low cost. Third, BECCS can provide a carbon negative energy source. Negative emission technologies (NETs) look attractive to the integrated assessment models because they effectively slow the required transition away from existing technologies, offsetting continued emissions in the short term with GHG removal in the longer term (Field & Mach, 2017). NETs are prominent in the second half of the century in most climate mitigation scenarios that achieve a 2 degree target and in virtually all scenarios that achieve the Paris goal of substantially below 2 degrees.

### 2.1 Baseload energy

Traditionally, power system planning involved designing the most cost‐effective mix of baseload electrical power (inflexible but cheap, such as coal or nuclear), load‐following power (which can adapt to daily or weekly variations in demand, but is more expensive), and peaking power (flexible but the most expensive, such as gas turbines). In that traditional arrangement, bioenergy was a logical alternative for baseload electrical power (along with nuclear, hydropower, and geothermal). Because decarbonization scenarios treat biomass as a low‐carbon and low‐cost fuel, it becomes an attractive alternative source of baseload power in those scenarios.

However, the declining prices of natural gas, solar, and wind power have fundamentally changed the approach to power system planning. Solar and wind power now have the lowest levelized cost of energy of any source of energy in two‐thirds of the world and will be the cheapest everywhere by 2030 (Bloomberg New Energy Finance, 2019). In 2019, the CEO of NextEra Energy, one of the largest power companies in the United States, stated that that solar and wind plus storage will be cheaper than coal, oil, or nuclear, and that this will be ‘massively disruptive to the conventional fleet’ (Roselund, 2019). As intermittent renewables make up a larger fraction of power, the need is no longer for baseload power that is rarely switched off, but instead for flexible, dispatchable power.

In a deeply decarbonized grid, flexibility will be required over different time frames, from minutes to seasons. A wide range of technologies and grid management strategies are available to meet this spectrum of flexibility needs. These include flexible sources of electrical power supply (e.g., gas, hydro), electricity storage (batteries, pumped hydro, compressed air), storage in chemical bonds (hydrogen production, synthetic fuels; Pierpont, Nelson, Goggins, & Posner, 2017), demand‐side measures (using prices to shift the timing of demand of industrial and residential customers), and improved integration of electric grid areas to provide increased flexibility (Schaber, Steinke, Mühlich, & Hamacher, 2012).

Where flexibility is needed over short time frames (minutes to hours), the declining cost of batteries means that by as early as 2030, battery storage will likely be cheaper than a new combined cycle gas turbine for providing intraday energy shifting (Polymeneas, Tai, & Wagner, 2018). But over longer time frames, intermittent renewables combined with storage are unlikely, at least over the next several decades, to be the most cost‐effective means to provide flexibility. At high penetrations of solar and storage it becomes difficult to replace the remaining natural gas or other ‘firm’ generation capacity with solar and storage without significantly overbuilding the solar or adding very long duration storage (Davis et al., 2018). For example, in a study of deep decarbonization for California, Ming, Olson, DeMoor, Jiang, and Schlag (2019) concluded that 17–35 GW of natural gas capacity would continue to be needed in 2050 even while reducing electricity sector emissions by 90%–95%, although the number of days when that capacity was used would decline significantly. Some studies suggest that ‘renewable hydrogen’ (generated from variable renewable sources via electrolysis) could provide an economically viable source of long‐term storage and a means of further decreasing the need for firm power (Element Energy, 2019), even though this technology is not yet the most cost effective means of producing hydrogen (Davis et al., 2018).

Thus, rather than being a substitute for ‘baseload’ power production, by mid‐century bioenergy will be competing with other energy sources to supply this firm power for interday and seasonal load balancing. For several reasons, it is unlikely that bioenergy will comprise a significant portion of this energy mix.

First, in some countries such as the United States, because of the low cost of natural gas, gas infrastructure is more pervasive and growing much faster than the infrastructure for bioenergy. Where that infrastructure exists, the least cost option for firm power will involve using existing natural gas power plants (at a reduced capacity factor) to avoid stranding those assets. Because they will be online for limited periods of time, their emissions will be relatively low. And, by mid‐century it is likely that these plants will use CCS or hydrogen. Already the United States provides a tax credit for power plants that capture and store CO_2_ and investments in new carbon capture technologies are growing.

For the bioenergy that is used for firm power, the most attractive source may not be land‐intensive biofuel. Instead, the use of biogas (a low‐carbon fuel produced from manure, municipal waste, and sewage) is likely to expand. For example, in 2016 biogas provided 17.2% of renewable fuel‐based electricity generation in Germany, only slightly less than PVs (Liebetrau, Denysenko, & Gromke, 2017). In contrast, by 2050 the large baseload powerplants that now burn wood pellets for fuel will not meet the flexibility needs of the future power grid because of the difficulty of ramping energy production to meet shifting demands.

Most importantly, as energy markets become more saturated with intermittent renewables, the economic incentive for affordable dispatchable power will grow significantly. While various types of bioenergy may be in the mix to meet that demand, they will be competing with a wide array of options including demand‐side responses, battery storage, hydro, concentrated solar power, power‐to‐gas, power‐to‐hydrogen, and natural gas with CCS. This situation will bear little resemblance to the traditional framing that substituted baseload coal power for baseload bioenergy under the assumption that it was the lowest cost low‐emission fuel.

### 2.2 Maritime and aviation fuels

For much of the transportation sector, the most promising energy source after mid‐century is likely to be electricity produced from low‐ or zero‐carbon sources. The International Energy Agency (IEA), for example, projects that for a scenario that aims to limit climate change to less than 2°C, by the mid‐2040s, nearly all two‐ and three‐wheelers and most passenger trains will be electric, and by 2060, around 90% of all cars on the road will be plug‐in electric (International Energy Agency, 2017a). But some modes of transportation, in particular the aviation sector, need much higher density fuels and so will be more difficult to electrify (particularly in the case of long‐haul flights; Davis et al., 2018). Jet fuel, for example, has an energy density nearly 50 times that of today's batteries.

Liquid biofuels are thought likely to be one of the most cost‐competitive sources of high energy density liquid fuels and have been seen as a promising replacement for maritime and aviation fuels and as a fuel that can help meet demands for any road transportation that is difficult to electrify. The IEA has projected that advanced biofuels will comprise 50% of the fuel mix for shipping and 70% of aviation fuel demand in 2060 (International Energy Agency, 2017a).

These projections assume that advanced biofuels will be the low‐cost, low‐carbon fuel, but there are other possibilities. By mid‐century, alternative zero‐carbon liquid fuels made from electricity or artificial photosynthesis (the utilization of light for splitting water into H_2_ and O_2_; Tachibana, Vayssieres, & Durrant, 2012) may be cost competitive with or even cheaper than biofuels. Detz, Reek, and Zwaan (2018), for example, performed a levelized cost analysis for seven routes to producing renewable fuels and found that after factoring in learning curves associated with individual system components, renewable energy pathways for generating both H_2_ and diesel fuel could be competitive with fossil fuels before mid‐century even in conservative scenarios. In more optimistic scenarios, renewable fuels were competitive with fossil fuels before 2030.

Other analysts are more cautious in their expectations about the potential for these technologies to begin to scale before mid‐century (Christensen & Petrenko, 2017; Schmidt & Weindorf, 2016). But whether renewable fuels begin to scale in 2040 or in 2070, the inefficient use of land and the inherent logistical challenges will make it difficult for land‐intensive bioenergy to be the long‐term fuel of choice in these sectors. Moreover, while the costs of alternative sources of energy generally decline as their production grows, it is unlikely that the costs of advanced biofuels based on land‐intensive bioenergy can decline as quickly as production grows because of land competition. Indeed, if any land‐intensive bioenergy gets to a significant scale, costs are likely to grow due to land competition.

The production of zero‐carbon (or carbon‐negative) renewable fuels will require zero‐carbon (or carbon‐negative) sources of electricity or artificial photosynthesis. To be most cost‐effective, these production systems would require a dedicated source of renewable electricity or hydrogen. But, with high penetration of intermittent renewables in electric grids, the production of renewable fuels will benefit during periods of surplus energy. In addition, a market for renewable fuels could have the significant cobenefit of helping to drive the development and scaling of Direct Air Capture (DAC; Friedmann, 2019; National Academies of Sciences, Engineering, & Medicine, 2018).

### 2.3 Bioenergy with CCS

Bioenergy with CCS is the most common NET in most mitigation scenarios today largely because it is arguably relatively low cost compared to technological approaches such as DAC and because some studies suggest that there is scope for a vast scale of deployment. But alternative technologies to BECCS (and afforestation) have not yet been comprehensively assessed in integrated assessment models (Rogelj et al., 2018), and no proposed NET technology is close to deployment at scale (Sanchez et al., 2019).

In fact, the assumptions that there is scope for a large‐scale of deployment of BECCS and that it will be the low‐cost NET at mid‐century are contested. The IPCC found that the average amount of BECCS in scenarios that achieve a 2 degree future would require 25%–46% of arable and permanent crop area to be devoted to bioenergy production in 2100 (de Coninck et al., 2018). We already face land scarcity today, and the IPCC concluded that land competition, uncertainties about the availability of water and nutrients, potential social conflicts, and the lack of public acceptability will pose constraints on the ability of BECCS to scale (de Coninck et al., 2018). A number of studies have concluded that biomass needed for high penetration of BECCS could not be supplied sustainably and that the trade‐offs with other land use would be high (European Academies Science Advisory Council, 2019; Fajardy, Köberle, MacDowell, & Fantuzzi, 2019; Field & Mach, 2017). Turner, Mach et al. (2018) estimate that the amount of BECCS that could be deployed on nonforested land that is not currently used for food production and that is overlying potential CO_2_ storage basins, amounts to only 10% of the amount that is typical in cost optimized model trajectories that stabilize warming at 2°C or less. And, the rates at which energy cropland expands to support BECCS in scenarios limiting temperature change to 2°C are exceedingly high, surpassing by more than threefold the observed expansion of soybean—the most rapidly expanding commodity crop (Turner, Field, Lobell, Sanchez, & Mach, 2018).

With regard to whether BECCS will be the least‐cost NET at mid‐century, in a 2018 review, Fuss et al. (2018) concluded that the best estimates for sustainable global NET potentials in 2050 for BECCS overlap with the estimates for DAC combined with carbon storage (BECCS: 0.5–5 Gt CO_2_/year at a cost of $100–$200/tCO_2_; DACCS: 0.5–5 Gt CO_2_/year at a cost of $100–$300/tCO_2_). Larsen, Herndon, Grant, and Marsters (2019) estimate that the first megaton scale DAC plant will have a cost of $124–$325/tCO_2_. When additional costs associated with pressurization and injection ($18/tCO_2_) and transportation are added, this would again overlap the mid‐century range of BECCS estimates. Already, the firm Carbon Engineering has calculated that CO_2_ could be removed from the atmosphere with its technology at a cost of $94–$232/tCO_2_ (Keith, Holmes, St. Angelo, & Heidel, 2018). And, the cost of BECCS, if deployed at a significant scale, is likely to increase with time given the pressures associated with the availability of land, while the cost of DACCS will decrease with time as the technology moves down the learning curve (Fuss et al., 2018).

In summary, for the three areas where most integrated assessment models anticipate high levels of use of bioenergy past mid‐century—baseload power, maritime and aviation fuels, and BECCS—the rising demand for biomass energy is probably mostly transient and should be a declining element of the long‐term energy mix after about 2050. This is due to three basic drivers:

- In each case, the costs of alternative technologies (e.g., storage, renewable fuels, DAC) appear likely to be competitive with land‐intensive bioenergy near mid‐century or earlier.
- While the costs of alternative technologies will continue to decline as they scale, significant scaling of land‐intensive bioenergy will likely result in increased costs due to land competition.
- Although models almost exclusively turn to bioenergy because of its low‐cost attributes, by 2050 the markets for firm power, transportation fuels, and NETs will inevitably foster a wide array of potentially competitive energy sources.

Given this transient role for land‐intensive biomass energy, what is the right balance between exploiting valuable energy resources and avoiding long‐term or even permanent losses in food security, valuable habitat, and biodiversity conservation?

## 3 BIOENERGY IN THE CURRENT CONTEXT

Although bioenergy may be a modest share of the 2100 energy mix, traditional and modern bioenergy currently accounts for 9.5% of primary energy supply (International Energy Agency, 2019). Both the amount and percentage of bioenergy are poised to grow significantly. Over the coming decades, demand for forest biomass is likely to increase as governments and power‐sector asset owners seek to maintain coal‐powered infrastructure while transitioning from coal. The production of wood pellets for biomass energy quadrupled to 26 million tons (MT) between 2006 and 2015 (Thrän et al., 2017). In the EU, which is the dominant importer of wood pellets, solid biomass accounts for nearly half (44.7%) of all renewable energy (40% of that biomass is used for residential heating). New biomass markets are also rapidly expanding in East Asia and could rival European demand in the near future. The government of Japan, for example, has approved 11.5 GW of biomass electricity projects (40% of which could be fueled by palm oil; Obayashi, 2017; Watanabe, 2017).

A key driver of this growth is the fact that the regulations in many countries treat biomass as a zero‐carbon fuel under carbon pricing regimes and for meeting national (and corporate) climate targets. This assumption results in greater use of bioenergy than is justified from a climate standpoint, since only a portion of the available biomass can provide a climate benefit over a 10 year time frame (European Academies Science Advisory Council, 2019). We use a 10 year time frame as being most relevant to actual climate impacts—if the use of bioenergy results in an increase in CO_2_ over a 10 year period, then it will exacerbate climate impacts, even if the regrowth of the fuel source eventually removes that carbon. Significant growth in the use of forest biomass has the potential to create a unique ‘double climate problem’ by simultaneously driving near‐term emissions greater than most fossil fuels, with long carbon payback periods of anywhere from decades to more than a century, and may degrade the ability of forests to fix carbon (Brack, 2017; Buchholz, Hurteau, Gunn, & Saah, 2016; Cornwall, 2017; Sterman, Siegel, & Rooney‐Varga, 2018).

With regard to biofuels, global biofuel production grew to 82 million tons of oil equivalent (MTOE) in 2017 and is projected to increase to 142 MTOE in 2040 (BP, 2019). In Indonesia, which has expanded its biofuels mandate from a 5% blend target in 2006 to 30% in 2020, potential growth in palm biodiesel demand could result in an additional 18.6 MT of palm oil demand by 2030 under high‐demand scenarios (Malins, 2017). Although Indonesia is some ways away from meeting its ambitious biodiesel blend targets, it currently uses only 35% of its existing palm biodiesel refining capacity, which suggests that production could increase substantially without major additional investments (Wright & Rahmanulloh, 2017). The combined demand for biofuels from these new and emerging markets has the potential to drive further deforestation in some of the world's last remaining intact forests, and increase carbon emissions in the transport sector (Malins, 2018; Meijaard et al., 2018).

Managing this awkward juxtaposition of likely near‐term growth in bioenergy use with the expectation of longer term decline raises a series of unique challenges. As a starting point, it is useful to consider three different categories of biomass supply, each of which can be extracted from ecosystems with different potentials and timelines for onsite and offsite carbon storage. Biomass can be a residue or waste product of other activities such as the production of timber or crops, or the use of cooking oil. Biomass can also be removed from ecosystems in order to increase carbon storage or improve the habitat in other ways. For example, biomass removal to reduce wildfire risk, increase tree growth, or facilitate increased utilization of wood fiber in long‐lived products can all increase carbon storage at the same time they provide a source for bioenergy. And, finally, biomass can be sourced from ecosystems that are managed specifically for energy (what we define as land‐intensive bioenergy). For each of these categories, the desirability, sustainability, and prospects differ substantially.

### 3.1 Waste biomass

Examples of waste biomass include waste wood from sawmills or small‐sized timber from logging operations, crop residues, and waste cooking oil. The use of waste biomass as an energy source to substitute for fossil fuels can be an effective mitigation strategy since these materials would decompose with time and lose carbon to the atmosphere in any event.

However, there are many industries (e.g., pulp and paper, construction, furniture, biorefinery) that compete for wood, and some other uses of these materials could be even better for climate. For example, the use of waste timber in the production of composite materials for building construction could lead to the long‐term sequestration of the carbon and offset high GHG emissions associated with the production of steel and concrete (Gustavsson et al., 2017).

Moreover, considerable care is needed in determining what is truly ‘waste’ biomass. Not all crop residues or slash from logging can be accurately characterized as waste, since the decomposition of these materials is important for the long‐term sustainability of these ecosystems (Liska et al., 2014; Vance et al., 2018). And, not all residues from logging operations would be expected to decompose rapidly—the payback periods for coarse residues could be several decades (Stenzel et al., 2019). Even in the case of used cooking oil, a growing international demand to use the oil for bioenergy could result in less reuse of oil in some regions, which would then be replaced by virgin oil.

While, in principle, pellets produced from waste biomass could provide net climate benefits compared to the use of fossil fuels, in practice the growing demand for biomass is rapidly exceeding the availability of waste biomass. The use of pellets, whether from waste or land‐intensive bioenergy, is also extending the life of coal‐fired power plants through cofiring (Bertrand, 2019), and there is a significant risk that the current trend toward coal‐to‐biomass conversions plus new biomass facilities will lock‐in large‐scale use of biomass for decades to come.

### 3.2 Good stewardship biomass

In certain circumstances, the carbon storage of natural ecosystems can be enhanced by the removal of biomass. For example, serious forest fires in the Western United States have resulted in significant greenhouse gas emissions. Forest thinning can help to reduce the risk of wildfires (Fulé, Crouse, Roccaforte, & Kalies, 2012) but results in significant removal of biomass. In another example, in South Africa, the Working for Water program seeks to restore landscapes by eradicating invasive alien plants. Stafford, Maltitz, and Watson (2018) found that the costs of the landscape restoration work could be substantially offset by the use of the invasive alien plant biomass for bioenergy. Similarly, in some grasslands, biomass removal increases the net primary productivity of the grassland (Yang, Tilman, Lehman, & Trost, 2018). In such cases, there is a carbon benefit from the removal of the biomass itself (as well as employment benefits associated with the forest thinning). Once biomass is removed, utilization for energy may be an appropriate fate, but as with waste products, incorporation into long‐lived products that replace GHG‐intensive alternatives may yield the best climate outcome.

In addition, in some cases biomass energy crops, particularly perennials, can help to improve soil quality in degraded lands and could provide an economically attractive means to begin to restore lands that would otherwise be extremely expensive to restore (Rahman et al., 2019; Tilman, Hill, & Lehman, 2006).

### 3.3 Dedicated biomass for energy

The sustainability and prospects for ecosystems managed for energy production are quite different for herbaceous crops, forest plantations, and naturally regenerating forests. Across all these systems, it can be challenging to operate in a mode that is truly climate beneficial, especially over a decade or less. After adjusting for emissions associated with transport and processing, indirect land use change, carbon debt, the ‘carbon opportunity cost’ of land converted to biomass production, and the potential to extend the lifespan of facilities that also burn fossil fuels through cofiring with biomass, some sources of bioenergy entail net emissions over a 10 year time frame that are worse than or comparable to the fossil sources they replace (European Academies Science Advisory Council, 2019; Searchinger, Wirsenius et al., 2018; Sterman et al., 2018; Zanchi, Pena, & Bird, 2012). Adding the pressures that land‐intensive bioenergy can place on food production (Frank et al., 2017) and biodiversity conservation (Smith & Torn, 2013) further tip the balance against sources that are marginally beneficial from a climate perspective.

Estimates of how much land‐intensive bioenergy could be sustainably produced in these three categories vary widely. Creutzig et al. (2015) concluded that there was relatively high agreement in the literature for a sustainable technical potential of up to 100 EJ/year, although the range spanned <50 to >1,000 EJ/year.

Because we see the issue of land scarcity and the importance of protecting and restoring ecosystem services to be a particularly important need, we would give higher weight to the more conservative estimates. For example, Field et al. (2008) estimate that ~27 EJ/year could be harvested from land that would not compete with food production (specifically, land that was previously used for agriculture or pasture but that has been abandoned and not converted to forest or urban areas). Canadell and Schulze (2014) developed similar estimates of bioenergy that could be produced with a high degree of environmental sustainability from largely abandoned agricultural lands and conclude that this would amount to between 26 and 64 EJ/year, equivalent to 3%–8% of the total primary energy by 2050, or 20%–40% of the median projections for bioenergy use in scenarios achieving a 1.5 degree target reviewed in Rogelj et al. (2018).

## 4 AVOIDING BIOENERGY LOCK‐IN

Historically, some resource‐intensive industries have faded only after the resource they relied on was largely or completely eliminated. Whaling and North‐American Bison hunting are classic examples, but fisheries, forests, and agriculture have been managed unsustainably in many places. In the 21st century, we have the potential to transform the planet at rates that were unimaginable until recently. And because land is absolutely fixed in quantity, land‐intensive bioenergy could transform lands at a scale and to an extent that are, in the absence of protections, fundamentally unacceptable. Given the indications that demand for bioenergy will fade over the course of the century, what are the kinds of protections that can build confidence in a sustainable future?

One lesson from history is that there are very few examples where governments or society have successfully orchestrated a smooth and economically efficient transition in any large‐scale complex system or industry, let alone an energy system. Instead, these systems typically exhibit path dependency, meaning that they develop inertial resistance to large‐scale systematic shifts, with resistance to change driven by favorable initial social and economic conditions and the momentum of increasing returns to scale (Seto et al., 2016).

Three factors contribute to the ‘lock‐in’ of an energy system (Seto et al., 2016). First, the lock‐in of physical infrastructure such as long‐lived power plants, pipelines, processing plants, buildings, and transmission systems could keep an energy system in place longer than would be optimal. Second, institutional lock‐in can reinforce the infrastructure lock‐in. Institutional lock‐in refers to economic, social, and political actors that seek to reinforce a status quo trajectory that favors their interests. As an industry develops, it gains economic and political power that it then uses to maintain the status quo, even when a transition would be better for society. Third, behavioral lock‐in, including social norms and cultural values, can further reinforce the status quo.

In the case of bioenergy, it is not hard to see the risks of physical, institutional, and behavioral lock‐in. If the expansion of energy crop production leads to the conversion of a natural ecosystem, it could take centuries to restore that ecosystem to its natural state. Already, wood pellets are being used to prolong the life of coal‐fired powerplants in Europe through cofiring or through a complete switch to biomass fuel, and dedicated biomass power plants are being built in Japan, South Korea, and the United Kingdom. These baseload powerplants, once built, will tend to slow the transition to energy systems that are more efficient in terms of cost or land‐use intensity. In the case of liquid biofuels, the US corn ethanol industry has gained tremendous political power that it has used to champion increases in the required blend of ethanol in gasoline despite the questionable benefits of corn ethanol for the climate.

Kalkuhl, Edenhofer, and Lessmann (2012) explore the economic implications of lock‐in of the electricity sector. They conclude that, especially given the high substitutability of alternative electricity sources, lock‐in of an inferior technology can persist for several decades, unless the lock‐in is addressed with specific policies. They found subsidies for the new technologies, feed‐in tariffs, and quotas for the legacy technologies to be effective tools for countering lock‐in.

To ensure that bioenergy meets near‐term needs for reductions in carbon emissions but can then transition to energy sources with better cost‐ and land‐use‐effectiveness, we will need policies to limit the infrastructural, institutional, and behavioral lock‐in. Even with that, path dependency is inevitable. What are the kinds of policies that can encourage appropriate expansion of bioenergy in the near term while also facilitating the transition to better technologies in the second half of the 21st century? Effective policies should account for the likelihood that strong economic and political forces that will help the industry scale in the next few decades will also tend to help the industry maintain or increase its market share after that.

A range of specific policies, certifications, and norms can help facilitate appropriate near‐term expansion of bioenergy while also discouraging inefficient lock‐ins in the longer term. Potentially effective policies fall into four broad categories. Some are focused on properly accounting for the benefits and costs of bioenergy, including recognition of the absolute constraint on land availability and on the prospects for restoration. Others focus on the characteristics of the feedstocks, avoiding those that are harmful to the climate over relevant time‐frames. Others nudge the biomass industry in the direction of avoiding commitments to long payback infrastructure. And still others incentivize the replacement technologies.

### 4.1 Policies to get the accounting right

#### 4.1.1 Complete accounting of GHG implications

**Figure 2:**
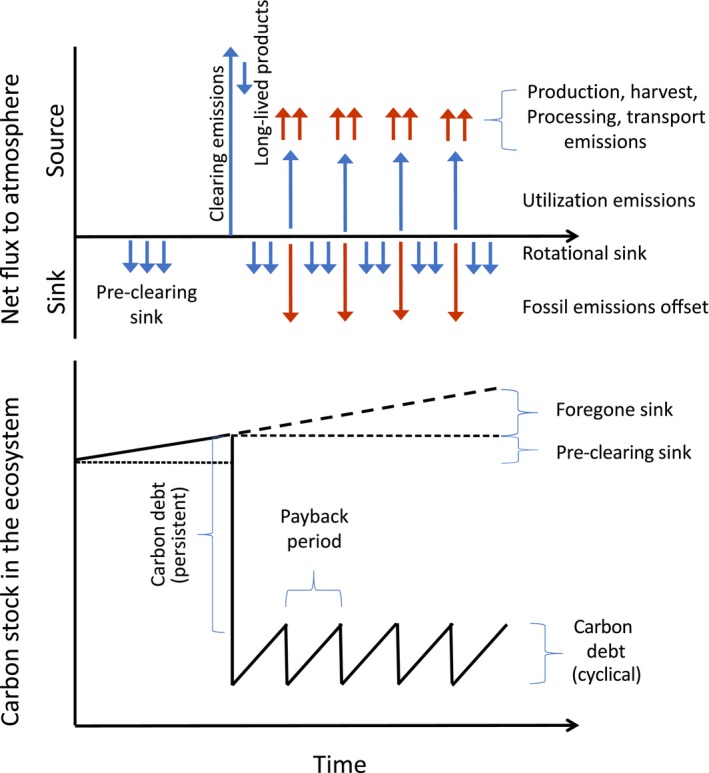
Schematic representation showing the elements of a complete GHG accounting system. For a full accounting of the GHG consequences of any biomass energy production system, it is essential to quantify: (a) emissions from production, harvesting, processing and transport, when these come from fossil fuels and not from harvested material; (b) emissions (persistent carbon debt) associated with the initial conversion from original vegetation to biomass crop. The carbon losses may occur over many years or decades as coarse woody debris and soil organic matter decompose. Harvested materials converted to long‐lived products should appear as offsets reducing the impacts of materials from the ecosystem; (c) rotational sources (cyclical carbon debt) and sinks (cyclical carbon repayment) that reflect changes in the site carbon balance with harvests over time. On a large landscape, these may be partly or completely smoothed; and (d) any foregone sink that would have operated in the absence of the initial clearing. Finally, fossil emissions offsets are an important component of the overall budget. Biogenic fluxes are shown in blue. Fossil fluxes are shown in red

GHG emissions from bioenergy can come from at least four factors. All four should be explicitly accounted in the GHG budgets for bioenergy, and any fossil offsets should be adjusted to reflect these emissions. They are (a) emissions from production, harvest, transport, and processing; (b) the carbon debt from converting any ecosystem into bioenergy production (although in select circumstances there could be a carbon gain, as for example could happen if degraded lands are planted with perennial bioenergy crops); (c) the payback period for a managed bioenergy landscape to return to preharvest carbon stocks following harvest of lands with large initial carbon stocks; and (d) loss of the counterfactual sink that would have occurred in the absence of a harvest, which would vary from relatively large in the case of forest ecosystems to relatively small for grassland ecosystems (Figure 2). Each of these has been discussed in the technical literature. One of the challenges in addressing the carbon debt and the payback period is that both have a time dimension not considered in most carbon accounting. One way to deal with the time dimension is to discount payback in the out years, as discussed by Newell and Stavins (2000) for forest sinks. A high discount rate can be a powerful disincentive for a strategy that counts on substantially delayed recovery of carbon stocks.

Complete accounting of GHG implications has two major benefits. The first is that credits for GHG reductions are consistent with what the atmosphere sees. The second is that, by aligning financial benefits for reducing GHG emissions with actual impact on emissions, high‐emission biomass energy is more likely to be passed over as noncompetitive. In addition, complete accounting of the GHG implications would tend to allocate forest biomass toward long‐lived wood products, where the GHG advantage over cement and steel is substantial.

In principle, complete accounting could be part of a voluntary certification system, utility regulations, national regulations, carbon pricing schemes, or international standards. However, a significant responsibility and accountability falls on the entity (i.e., power plant, nation, emission trading system) reporting the emissions or benefiting from a fossil offset. For a country to claim an emission reduction associated with biomass imported from another country, for example, it would need to have a mechanism in place to ensure that there is no double‐counting between the countries and that the carbon impacts associated with the biomass supply are appropriately accounted (Schneider et al., 2019).

Complete GHG accounting should largely prevent the use of whole trees or logs from existing plantations to be used for bioenergy since the long payback period will lead to an increase of GHG emissions compared to fossil fuels over a period of decades. The time pressure of getting emissions under control argues that future increases in onsite carbon stocks should be discounted at a rate that reflects the urgency of the need for emissions reductions.

This does not mean that it is impossible for whole trees to be harvested as a source of low‐emission bioenergy. For example, the current long‐term and large‐scale system of forest management and harvest rotation in Sweden, and the use of that wood to offset the use of fossil fuels, is effective as a climate mitigation strategy (Egnell, Ahlgren, & Berndes, 2018). More generally, over a large enough scale and over a long enough period of time, bioenergy production that would initially result in a net increase in CO_2_ in the atmosphere will become steadily lower in net emissions. However, our concern in this paper is what the atmosphere will see in the next several decades, which is the crucial time frame we have to address climate change, and over that time frame the near‐term additions of CO_2_ are harmful even if the system becomes more sustainable with time.

#### 4.1.2 Land adjuster for GHG accounting

Unregulated markets are less than ideal mechanisms for allocating land. Some kinds of land uses fit comfortably in markets, but many do not. Market prices are mostly not relevant for land for conservation, habitat, recreation, air and water quality, indigenous peoples, and subsistence farmers, even though pricing of natural capital provides an increasingly established and sophisticated portfolio of frameworks and techniques (Daily et al., 2000). One way to address the distortions caused by the absence of multidimensional markets for land is to institute a land adjuster for GHG accounting. With such an adjuster, energy sources are penalized for the land they occupy, and the penalty would be largest for technologies that require the largest amount of land per unit of energy produced.

**Figure 3:**
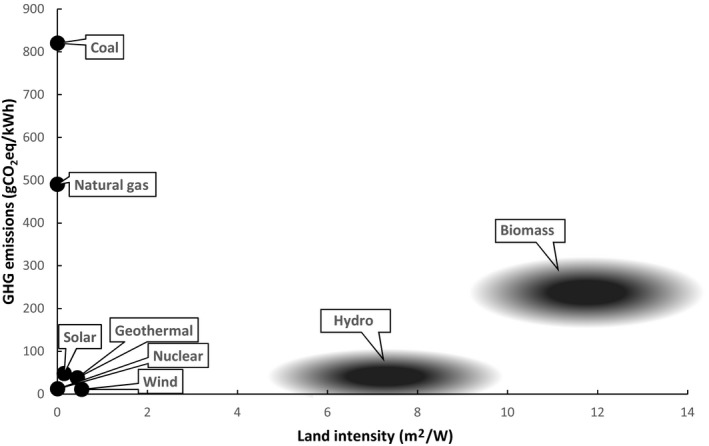
Relationship between median GHG emissions per unit energy for different fuels and median land area per unit energy required for the production of each fuel. Median lifecycle g CO_2_eq/kWh from IPCC (2014) and median m^2^/W from van Zalk and Behrens (2018)

The adjuster for example could take the form of a per hectare fee charged for uses of land for energy production. A solar or wind installation, with its power density (W/m^2^) one to two orders of magnitude higher than bioenergy, would thus pay a much smaller fee than a bioenergy facility (Figure 3). A natural gas facility would face an even smaller land adjuster fee but would have far higher emissions than solar or wind. The adjuster could take different values for, for example, old growth forest, cropland, rangeland, and desert. The implementation would be different depending on whether the overarching policy regime is a carbon price, a renewable portfolio standard, an offsets market, or a voluntary certification system. But for every implementation, the adjuster would be structured to provide a low penalty for land‐efficient energy and a substantial penalty for land‐intensive energy. Raising the adjuster in the second half of the century could be a powerful tool for calibrating the relative values of competing uses for land.

### 4.2 Favoring biomass from waste and ecosystem improvement

Forest bioenergy can deliver meaningful climate benefits over the next 50 years under three classes of conditions: (a) when the source of the biomass is waste left over from other operations; (b) when the goal of the biomass removal is improving the ecosystem through, for example, wildfire risk reduction; or (c) when biomass is grown on land with low‐carbon stocks that would otherwise remain unused. Although it has been argued that more widespread use of forest biomass for bioenergy would create a market incentive that would lead to an overall increase in forest biomass through improved forest management and new plantings, a recent review of forests in Canada, Sweden, and the United States does not support that conclusion (Giuntoli & Searle, 2019). Ensuring that bioenergy is in fact sourced from these three categories would require a full chain‐of‐custody tracking system that does not yet exist in any country.

In general, no bioenergy should be obtained from harvesting naturally regenerating forests, except as waste for site improvement. Careful accounting of the carbon debt and payback period should generally assure this, though a specific rule might provide valuable clarity and simplicity.

### 4.3 Avoiding investments in long‐lived bioenergy infrastructure

Countries could reduce the risk of lock‐in of bioenergy facilities by limiting investments in new baseload power plants using biomass. They should similarly avoid subsidizing the construction of new biofuel infrastructure to produce biofuels for bunker fuels and jet fuels. These assets are likely to be stranded as renewable penetration advances. Because of the long service life of major infrastructure, construction in the next decade will produce incentives to continue to use that infrastructure even though society and the climate would be better off with a more rapid transition.

### 4.4 Incentivizing replacement technologies

As discussed by Kalkuhl et al. (2012), incentives favoring new technologies can be effective tools in minimizing lock‐in from legacy technologies. They found that subsidies, feed‐in tariffs, and quotas can all be close to economically optimal. At this point, the greatest need for incentives is probably not in the area of electricity generation but more in the areas of energy storage (especially long‐term, large capacity), electricity transmission, and electrified heavy transportation. In the case of energy used for heating, there is an ongoing need for research and development to scale low‐carbon alternatives to coal and biomass fuels. There is a continuing need for incentives to encourage the electrification of the light duty vehicle fleet. And, incentives are needed to promote the further development of DAC technologies which can play an important role in negative emissions. In all these cases incentives could take several different forms, including research investments, tax credits, and government procurement policies.

All these policies and norms could be designed to be more permissive or more conservative, and all can be adjusted over time. The fixed size of the land estate argues for a conservative approach, especially since many ecosystem features are difficult or impossible to restore once lost.

If fully and effectively implemented, policies adhering to these norms would both ensure that the bioenergy used at any point in time is contributing to climate mitigation (Sections 4.1 and 4.2 above) and substantially reduce the risks of economically and environmentally harmful path dependency (Sections 4.3 and 4.4 above). For example, if there were complete accounting of GHG implications (Section 4.1) then the common practice of treating bioenergy combustion emissions as zero‐carbon would be reasonable because the associated carbon emissions would appear on an accounting ledger as: (a) energy related emissions associated with the production and transport of the pellets, and (b) a loss of carbon in the ecosystem where the pellets originated. If the ledger accurately showed net CO_2_ emissions, then appropriate policies could provide the incentives needed to reduce those emissions. While such a system of complete accounting combined with effective accountability policies and incentives is possible, it does not exist today. And, in many countries, it would be difficult to fully and effectively implement such policies. For many countries the data and monitoring systems required are beyond their current capacity, and protocols have not been developed that account for land use carbon changes associated with bioenergy use, particularly when biomass is burned for energy in a different country from where it was produced.

Given that reality, are there policies that do not require such complete accounting but assure outcomes that approximate adherence to these norms? While we see the development of the more accurate accounting framework to be desirable, a regulatory approach based on more readily obtained information could result in similar outcomes. Some examples of such policies include regulations that:

- Prevent the use of bioenergy obtained from harvesting naturally regenerating forests. This bioenergy would inevitably have a long payback time and erode ecosystem services.
- Prevent the conversion of naturally regenerating forest to dedicated bioenergy plantations, unless the forests are on degraded land, are dead or dying, or at a high risk of loss from wildfire. Again, this bioenergy would have a long payback.
- Alter any policy or standard that considers biomass to be a zero emissions feedstock to one based on a best‐practices complete accounting. This would provide important resistance to incentives that inappropriately favor wood pellets for electricity and heat.
- Prevent the expansion of biofuels produced from food crops or from the conversion of food cropland to biofuel production. Land is already scarce and expanding the use of crop land for biofuels will require additional clearing to produce food, resulting in both increased GHG emissions and further loss of ecosystem services and biodiversity.

## 5 CONCLUSION

Land‐intensive bioenergy is already a significant part of the global energy mix. Based on current trends and policies, it is likely to increase in the next decade or more. But the scale of bioenergy that both provides net climate benefits and can be sustainably produced is more limited than most models and scenarios predict. It is unlikely that land‐intensive bioenergy will be a significant part of the energy mix by the end of the century. Policymakers should have the following goals in mind as they consider the use of bioenergy in the coming decades. First, any use of bioenergy as a substitute for fossil fuels should result in a significant reduction of emissions over the short time periods (years rather than decades) that matter for climate impacts. When biomass is available as a waste product or as a result of good stewardship practices, the best use of the material is for long‐term storage as for example in the construction of buildings. The second‐best use is for energy and only if that energy production does not create problems associated with air or water pollution or water scarcity (and ideally if it is equipped with CCS). Land‐intensive bioenergy is likely to be seen as a legacy fuel by mid‐century. In light of this, policymakers should limit near‐term incentives for land intensive bioenergy and instead should provide incentives for the next generation of technologies that will allow a zero‐carbon future.
